# Are we ready to stratify BI-RADS 4 lesions observed on magnetic resonance imaging? A real-world noninferiority/equivalence analysis

**DOI:** 10.1590/0100-3984.2023.0087

**Published:** 2023

**Authors:** João Ricardo Maltez de Almeida, Almir Galvão Vieira Bitencourt, André Boechat Gomes, Gabriela Lemos Chagas, Thomas Pitangueira Barros

**Affiliations:** 1 CAM – Clínica Médica e Imagem (Grupo Oncoclínicas), Salvador, BA, Brazil; 2 A.C.Camargo Cancer Center, São Paulo, SP, Brazil.

**Keywords:** Breast neoplasms, Magnetic resonance imaging, Radiology information systems, Breast/diagnostic imaging, Predictive value of tests, Neoplasias da mama, Ressonância magnética, Sistemas de informação em radiologia, Mama/diagnóstico por imagem, Valor preditivo dos testes

## Abstract

**Objective:**

To demonstrate that positive predictive values (PPVs) for suspicious (category 4) magnetic resonance imaging (MRI) findings that have been stratified are equivalent to those stipulated in the American College of Radiology Breast Imaging Reporting and Data System (BI-RADS) for mammography and ultrasound.

**Materials and Methods:**

This retrospective analysis of electronic medical records generated between January 4, 2016 and December 29, 2021 provided 365 patients in which 419 suspicious (BI-RADS category 4) findings were subcategorized as BI-RADS 4A, 4B or 4C. Malignant and nonmalignant outcomes were determined by pathologic analyses, follow-up, or both. For each subcategory, the level 2 PPV (PPV2) was calculated and tested for equivalence/noninferiority against the established benchmarks.

**Results:**

Of the 419 findings evaluated, 168 (40.1%) were categorized as malignant and 251 (59.9%) were categorized as nonmalignant. The PPV2 for subcategory 4A was 14.2% (95% CI: 9.3–20.4%), whereas it was 41.2% (95% CI: 32.8–49.9%) for subcategory 4B and 77.2% (95% CI: 68.4–84.5%) for subcategory 4C. Multivariate analysis showed a significantly different cancer yield for each subcategory (*p* < 0.001).

**Conclusion:**

We found that stratification of suspicious findings by MRI criteria is feasible, and malignancy probabilities for sub-categories 4B and 4C are equivalent to the values established for the other imaging methods in the BI-RADS. Nevertheless, low suspicion (4A) findings might show slightly higher malignancy rates.

## Abstract (pt)

**Objetivo:**

Demonstrar que os valores preditivos positivos (VPPs) para lesões suspeitas (categoria 4) identificadas por ressonância magnética (RM) são equivalentes aos estipulados no ACR BI-RADS para mamografia e ultrassonografia.

**Materiais e Métodos:**

Análise retrospectiva de dados em prontuário eletrônico, entre 4 de janeiro de 2016 e 29 de dezembro de 2021, resultou em 365 pacientes elegíveis, com 419 lesões classificadas como BI-RADS 4A, 4B ou 4C. Desfechos malignos e não malignos foram determinados por estudo patológico e/ou acompanhamento. Realizamos o cálculo do VPP nível 2 (VPP2) para cada subcategoria e testamos para não inferioridade/equivalência em relação aos valores de referência.

**Resultados:**

Dos 419 achados, 168 (40,1%) foram malignos e 251 (59,9%), não malignos. O VPP2 para subcategoria 4A foi 14,2% (IC 95%: 9,3–20,4%), para 4B foi 41,2% (IC 95%:, 32,8–49,9%) e para 4C foi 77,2% (IC 95%: 68,4–84,5%). Análise multivariada demonstrou diferenças estatisticamente significantes entre as subcategorias (*p* < 0,001).

**Conclusão:**

A estratificação de achados suspeitos por RM é factível, sendo que a probabilidade de malignidade das subcategorias 4B e 4C é equivalente à estabelecida para outros métodos de imagem pelo BI-RADS. Contudo, lesões de baixa suspeição (4A) podem apresentar taxas mais altas de malignidade.

## INTRODUCTION

Breast cancer is the most prevalent malignancy in women worldwide (excluding nonmelanoma skin cancers) and has shown an increasing trend in many high-income countries^(1)^. Nevertheless, since the 1980s, mortality rates have been steadily declining because of a variety of factors, one being the widespread implementation of secondary prevention programs, mainly through mammographic screening^(2,3)^. Over time, ultrasound and magnetic resonance imaging (MRI) became more widely available and developed into valuable complements to mammography. MRI soon came to be recognized as a screening method for high-risk women (those with a lifetime risk of 20–25% or greater), adopted by most international medical societies, with ever increasing recommendations due to its unparalleled sensitivity and potential to better characterize breast malignancies^(4,5,6,7)^. It has been a part of the American College of Radiology Breast Imaging Reporting and Data System (ACR BI-RADS) since its fourth edition^(8,9)^.

The ACR BI-RADS is considered a “living” document, and its many sections include an imaging lexicon, assessment categories, recommendations for practice, and general tools for quality auditing^(10,11)^. One of its many goals is to integrate varied breast imaging methods, providing coherent terminology and concordant categories according to the malignancy probability of the observed findings. MRI is the most recent modality included in the BI-RADS, and because of its technical particularities and scarcity of data pertaining to specific topics in cancer detection, it has yet to be fully integrated into the BI-RADS corpus^(12,13)^. One issue that stands out in its most recent edition is the lack of defined criteria for the stratification of suspicious (category 4) MRI findings^(8)^.

The wide range of malignancy probabilities encompassed by BI-RADS assessment category 4 (> 2% and < 95%) confuses patients and poses a potential problem to assisting physicians^(14,15,16)^. To address the matter, the two latest editions of the BI-RADS stratified suspicious lesions found on mammography and ultrasound, but not those found on MRI, into three subcategories, by malignancy probability^(8,15)^: 4A (> 2% and ≤ 10%); 4B (> 10% and ≤ 50%); and 4C (> 50% and < 95%). The outcome of this approach influences clinical practice, given that it improves the radiologic-pathologic correlation, which can preclude the need for ongoing invasive studies in cases with a low suspicion for malignancy^(17,18,19)^. The importance of stratifying suspicious MRI findings cannot be underestimated, given that MRI-guided procedures are not widely available and, in most parts of the world, are considered financially out of reach for the general population^(20,21,22)^.

This study investigates whether category 4 stratification by MRI criteria, based on the accepted descriptors, is equivalent/noninferior to that already established for mammography and ultrasound in the ACR BI-RADS.

## MATERIALS AND METHODS

### Study subjects

This retrospective study was analyzed and approved by an independent review board from one of the sponsor institutions. Because of the retrospective nature of the study, the requirement for informed consent was waived.

**Figure 1:**
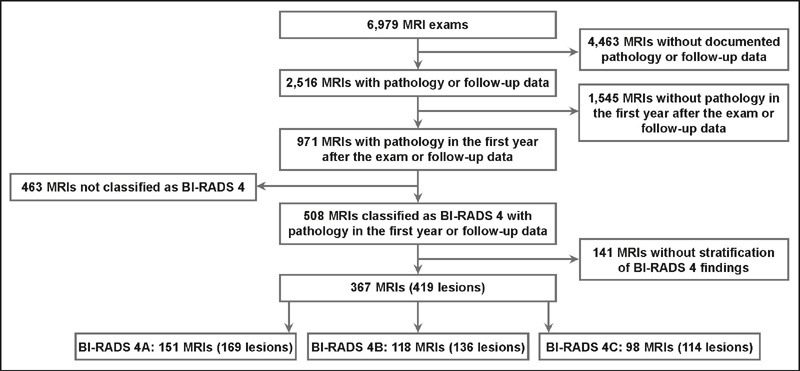
Flow chart of the selection of stratified suspicious (category 4) findings, yielding 419 eligible lesions from 367 examinations (in 365 patients).

We executed a stepwise computerized search of the anonymized electronic database of our institution, which is a regional private referral center for breast cancer. We included all consecutive breast MRI studies performed between January 4, 2016 and December 29, 2021, regardless of their indication. Of the 6,979 breast MRI examinations included, 2,516 (36.05%) prompted invasive investigation (defined as any kind of needle aspiration/biopsy or surgery), at any time, or were in patients who were followed for at least three years, as documented in our records. To narrow the search and minimize the number of unrelated breast biopsies, we looked for subjects who had undergone invasive procedures only in the first year after MRI, thus obtaining 971 examinations. Next, excluding repeat examinations without new suspicious findings (examinations that showed new lesions were included), as well as cases in which biopsies unrelated to the BI-RADS 4 lesion were performed, reduced the number of breast MRIs to 508 (52.32% of the 971). We then excluded 141 studies in which the findings were not subcategorized. Therefore, the final sample comprised 367 examinations (72.24% of the 508) in 365 patients (two had new findings in subsequent examinations during the study period), among which a total of 419 lesions were subcategorized as low, moderate, or high suspicion for malignancy (4A, 4B, and 4C, respectively). Figure 1 illustrates the selection process.

### Breast MRI technique

The studies were performed in three different 1.5-T MRI suites—one with a Signa Excite HDxT scanner (upgraded to HD23) and two with Optima 360 scanners—all from GE Healthcare (Milwaukee, WI, USA). Because all of the scanners were from the same vendor, similar protocol parameters could be applied to them.

All of the scanners use eight-channel bilateral phased-array breast coils, and we began with a three-plane localizer, followed by three sets of acquisitions in the sagittal plane. The specifications for the Signa HD23 are as follows: the first acquisition is a T1-weighted fiast spin-echo sequence—repetition time/echo time (TR/TE), 400/15 ms; echo-train length, 5; bandwidth, 41.7 MHz; number of signals averaged (NSA), 1; matrix size, 320 × 224; field of view (FOV), 200 × 200 mm; slice thickness, 4 mm; interslice gap, 0.5 mm—which is followed by a fat-suppressed T2-weighted sequence—TR/TE, 4,500/85 ms; echo-train length, 17; bandwidth, 25.0 MHz; NSA, 3; matrix size, 256 × 192; FOV, 200 × 200 mm; slice thickness, 4 mm; interslice gap, 0.5 mm—and a set of three-dimensional (3D) fast spoiled gradient-recalled echo sequences, with parallel volume imaging for breast assessment (VIBRANT) in the sagittal plane as the dynamic study, one sequence before contrast media injection and three after (TR/TE, 5.5/2.7 ms; flip angle, 15°; bandwidth, 50.0 MHz; NSA, 1; matrix size, 320 × 192; FOV, 200 × 200 mm; slice thickness, 3 mm; interslice gap, 0 mm; reduction factor, 2). Next, we acquired a single late-phase contrast-enhanced 3D VIBRANT sequence (TR/TE, 5.0/2.4 ms; flip angle, 15°; bandwidth, 62.5; NSA, 1; matrix size, 350 × 350; FOV, 340 × 340 mm; slice thickness, 1 mm; interslice gap, 0 mm; reduction factor, 2).

On the Optima 360 scanners, all parameters were kept the same as those used on the Signa HD23 scanner, except for the following: slightly longer TR and shorter echo-train length on the fat-suppressed T2 sequence (TR, 4,900 ms; echo-train length, 5), and longer TR/TE on the VIBRANT acquisition (TR/TE, 6.4/2.7 ms), with a slightly smaller matrix and FOV (matrix, 288 × 192; FOV, 200 × 200 mm).

Up until 2017, we used gadoterate meglumine (Dotarem; Guerbet, Roissy, France). Since then, we have been using gadobutrol (Gadovist; Bayer Schering Pharma AG, Berlin, Germany), applying 0.1 mmol/kg of body weight as a bolus injection, followed by a 20 mL saline flush.

### Image analysis and data collection

**Figure 2:**
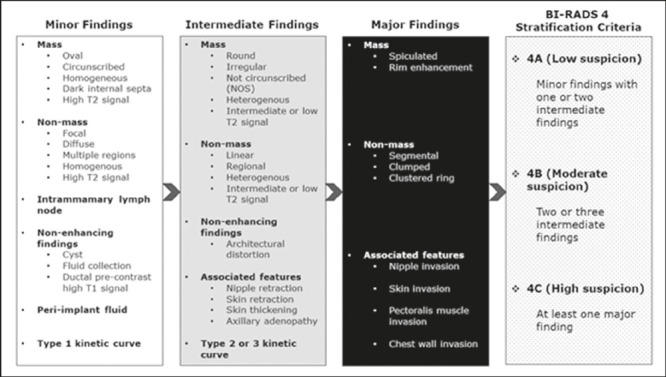
MRI criteria derived from ACR BI-RADS descriptors. In order to be considered suspicious, a lesion must have at least one intermediate finding (gray box) related to mass or non-mass enhancement. The findings are additive and progressively upgrade BI-RADS 4 subcategories, as shown in the box at the extreme right (dot-pattern box). Non-enhancing findings and associated features, when present, might also upgrade the BI-RADS 4 subcategory of the lesion, but should not be considered in isolation without enhancing abnormalities. There is some overlap between the number of intermediate findings observed in subcategories 4A and 4B, allowing for the personal experience of the examiner, given that there are no established MRI criteria in the ACR BI-RADS.

**Figure 3:**
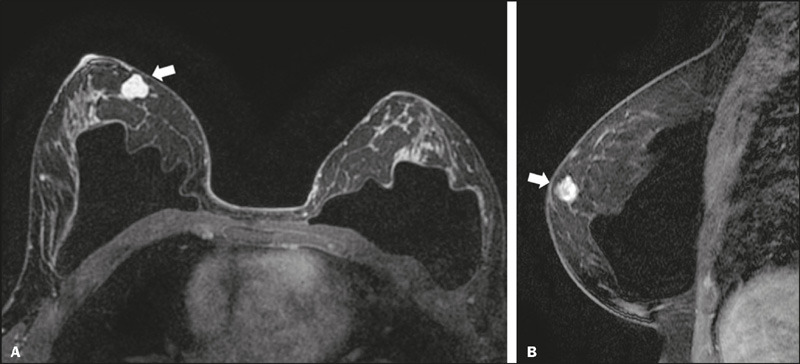
A 31-year-old female with a mass found in the right breast on ultrasound (not shown) was submitted do breast MRI. Axial and sagittal contrast-enhanced T1-weighted images (**A** and **B**, respectively) showing a round mass described as having “slightly irregular margins” (arrows) and classified as low suspicion—BI-RADS 4A. After ultrasound-guided core needle biopsy, the mass was diagnosed as a fibroadenoma.

**Figure 4:**
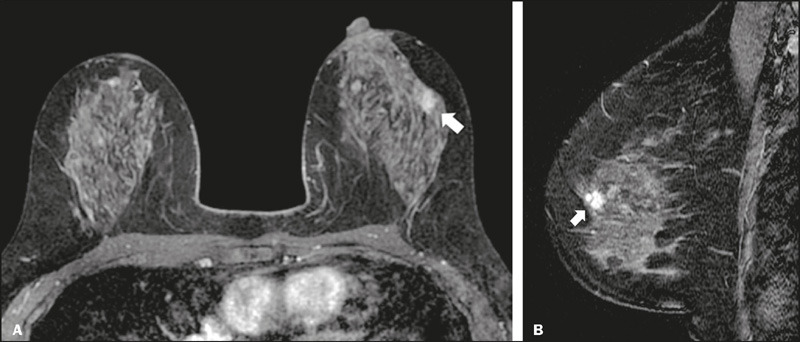
A 76-year-old female with non-mass enhancement in the right breast. Axial and sagittal contrast-enhanced T1-weighted images (**A** and **B**, respectively) demonstrating a “regional, heterogeneous” area of enhancement (arrows), classified as moderate suspicion—BI-RADS 4B. The lesion was surgically excised and diagnosed as invasive lobular carcinoma.

**Figure 5:**
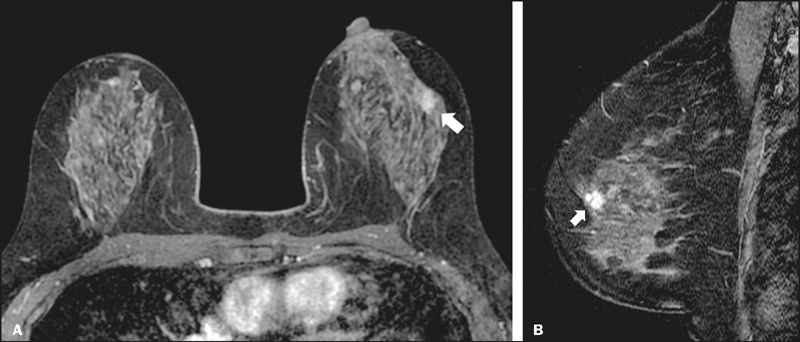
A 48-year-old patient was found to have a suspicious nodule on routine screening. Axial and sagittal contrast-enhanced T1-weighted images (**A** and **B**, respectively) showing a “spiculated mass” in the left breast (arrows), classified as high suspicion—BI-RADS 4C.

All breast MRI studies were interpreted as part of the daily workload of a typical radiology clinic and were reported according to directions found in the fifth edition of the ACR BI-RADS^(8)^. Three radiologists, working independently, interpreted the images using information about previous examinations and the clinical data available. Two of the radiologists had more than ten years of experience in the field of breast MRI, and one had more than five years of experience in the same field. At our institution, despite the lack of official ACR BI-RADS recommendations for MRI, it is common practice to stratify category 4 MRI lesions by means of personal experience based on published guidelines and positive predictive values (PPVs) for specific descriptors^(23,24)^. A guide to our stratification criteria can be seen in Figure 2. We consider the primary characteristics related to mass and non-mass enhancement, adding the observed descriptors to determine the BI-RADS 4 subcategory. Non-enhancing and other associated features, if present, might upgrade the stratification but typically are not to be considered in isolation. There is some intended overlap between the number of descriptors used to stratify 4A and 4B lesions (Figures 3 and 4, respectively), allowing subjective judgment based on the clinical context and, in some cases, on additional information from other evaluations that were available to the radiologists (including a family history of breast cancer, mammography results, ultrasound findings, and other relevant information). In contrast, descriptors with higher predictive values would be necessary for classifying any finding as subcategory 4C (Figure 5), resulting in less subjectivity. The interpreters were free to stratify only the cases they considered appropriate, although they stratified all of those for which stratification was explicitly requested by the ordering physicians.

Over the course of the study period, all of the images were initially evaluated with different versions of the same visualization tool (RadiAnt DICOM Viewer, from version 2.29, December 27, 2015 up to version 2021.2, October 24, 2021; Medixant, Poznan, Poland; https://www.radiant-viewer.com). At the discretion of the examiner, the images were further analyzed on a vendor-specific workstation (Advantage Windows, version 4.4; GE Healthcare).

### Pathology and follow-up

Pathology results, with or without at least three years of follow-up data, were recorded in our electronic medical records for all eligible cases. Most of the patients had undergone more than one invasive diagnostic procedure, ranging from fine-needle aspiration to surgical excision. Whenever fine-needle aspiration was performed, further pathological investigation was left to the discretion of the attending physician. Nevertheless, discordant, inconclusive, or suspicious cytopathology findings necessarily led to further investigation with tissue sampling, except when the patient did not agree to undergo the procedure, opting for closer follow-up.

For any abnormality that did not clearly correlate with other imaging methods, a second-look ultrasound was initially recommended. Lesions that were MRI-exclusive were referred for MRI-guided procedures. Because of limited availability and financial concerns, the biopsies and localizations were guided by ultrasound or mammography whenever a feasible correlation with the MRI findings could be achieved.

The most conclusive pathology report available (e.g., the result of a cytopathology study followed by a tissue sampling procedure and histopathological analysis classified according to the latter) was used as the defining outcome. Whenever mixed histopathological abnormalities were described, the most aggressive or dominant finding would determine in which group the subject would be placed (e.g., findings of atypical ductal hyperplasia and invasive ductal carcinoma would be considered indicative of malignancy). The final dichotomous outcome analysis grouped the findings as nonmalignant (including typically benign, indeterminate, and high-risk lesions) or malignant (including ductal carcinoma *in situ* and any type of invasive carcinoma). All lesions of indeterminate or high-risk pathology, as determined by tissue sampling, were designated for further surgical excision.

### Statistical analysis

We included in our analysis the ages of the patients, the total number of category 4 lesions, and their stratification as 4A, 4B, or 4C. Categorical variables are expressed as absolute and relative frequencies, together with 95% confidence intervals (95% CIs) when applicable, whereas the one continuous variable (age) is expressed as median, range, and interquartile range (IQR). Fifty-four subjects had more than one suspicious lesion during the study (two patients had novel findings in consecutive examinations). Therefore, the mean number of lesions per patient was 1.15. Because the number of observations per patient was considered small, resulting in a very small kappa and in-tracluster correlation coefficient, we reported the original statistical test results, considering a per-lesion analysis, without applying any correction factor^(25)^.

The Mann-Whitney U test was applied to determine whether there was a significant age difference between the malignant and nonmalignant groups. We employed the chi-square test and Fisher’s exact test with Bonferroni correction, when applicable, to examine the categorical outcomes, particularly if the BI-RADS 4 stratification levels were related to different malignancy frequencies. For all of the category 4 findings and for each subcategory, we calculated the level 2 PPV (PPV2), from which we derived the 95% CIs by the Clopper-Pearson method.

In order to determine whether the PPV2 results were equivalent/noninferior to those published in the ACR BI-RADS fifth edition, we compared them and their 95% CIs to the recognized parameters for mammography and ultrasound (malignancy probability from > 2% to ≤ 10% for subcategory 4A; from > 10% to ≤ 50% for subcategory 4B; and from > 50% to < 95% for subcategory 4C). The PPV2 outcomes were considered equivalent only when their 95% CIs were between the established percent margins for each subcategory. Whenever the PPV2 was in the equivalence zone but one or more of the bounds of the 95% CIs (upper, lower, or both) crossed the margins, the result was considered inconclusive. If the PPV2 was outside the equivalence zone, it was considered nonequivalent, even if the 95% CIs breached the equivalence margins^(26)^.

Finally, we generated a multivariate logistic regression model to predict malignancy probabilities by age and BI-RADS 4 subcategory. These variables would be included only if the *p*-value was below 0.10 in the univariate analyses, which would lead to a backward stepwise conditional insertion into the multivariate model. We reported crude odds ratios (ORs) and 95% CIs, assessed the fit of the model using the Hosmer-Lemeshow goodness-of-fit test. Then we used the full model probabilities of malignancy to generate a receiver operating characteristic (ROC) curve and to calculate the area under the curve (AUC). All calculations were performed in the IBM SPSS Statistics software package for Windows, version 21.0 (IBM Corp., Armonk, NY, USA), and a two-tailed value of *p* < 0.05 was considered statistically significant.

## RESULTS

### Subjects and lesions

During the study period, 367 MRI examinations were carried out in 365 subjects, revealing 419 suspicious findings that were stratified as BI-RADS 4A, 4B, or 4C. On the basis of the pathological analysis and clinical follow-up data, 168 (40.1%) of the 419 findings were classified as malignant and 251 (59.9%) were classified as nonmalignant. As can be seen in Table 1, 228 (90.8%) of the 251 lesions in the nonmalignant group were typically benign pathologic abnormalities (accounting for 54.4% of the sample as a whole) and 23 (9.2%) were of an indeterminate or high-risk nature (accounting for 5.5% of the sample as a whole). Of the 419 findings evaluated, 383 (91.4%) were the target of at least one tissue sampling procedure and 36 (8.6%) were subjected only to cytopathology and clinical follow-up because the cytopathology findings were indicative of a benign lesion. Therefore, the cancer yield differed significantly between the cytopathology and histopathology reports (Fisher’s exact test, *p* < 0.001), as shown in Table 2. Patient ages ranged from 22 to 96 years (median, 50 years; IQR, 42–61 years), with median ages in the non-malignant and malignant groups of 48 years (IQR, 41–58 years) and 56 years (IQR, 46–65 years), respectively, the difference between the groups being significant (U, 11,556.50; *p* < 0.001).

**Table 1:** Pathology results for the lesions evaluated (N = 419).

Pathology	n(%)
Nonmalignant (benign)	228 (54.4)
Fibroadenoma	33 (7.9)
Papilloma (without atypia)	33 (7.9)
Stromal fibrosis	27 (6.4)
Adenosis/sclerosing adenosis	24 (5.7)
Fibrocystic changes	23 (5.5)
Usual ductal hyperplasia	16 (3.8)
Benign (not otherwise specified) or negative for cancer	14 (3.3)
Pseudoangiomatous stromal hyperplasia	10 (2.4)
Normal tissue	10 (2.4)
Mastitis	8 (1.9)
Lymph node	8 (1.9)
Fat necrosis	7 (1.7)
Limited cytology sample	5 (1.2)
Cyst	4 (1.0)
Abscess	3 (0.7)
Systemic disease (nonmalignant)	2 (0.5)
Flat epithelial atypia	1 (0.2)
Nonmalignant (indeterminate to high risk)	23 (5.4)
Complex sclerosing lesion	13 (3.1)
Atypical ductal hyperplasia	4 (1.0)
Papilloma with atypia	2 (0.5)
Atypical lobular hyperplasia	1 (0.2)
Adenosis with atypia	1 (0.2)
Intracystic papillary growth	1 (0.2)
Fibroepithelial neoplasia not otherwise specified	1 (0.2)
Malignant	168 (40.1)
Invasive ductal carcinoma	100 (23.9)
Ductal carcinoma in situ	44 (10.5)
Invasive lobular carcinoma	19 (4.5)
Mucinous carcinoma	3 (0.7)
Phyllodes tumor	1 (0.2)
Metastasis to the breast	1 (0.2)

**Table 2:** Sampling methods and their respective cancer yields for the lesions evaluated.

Sampling method	All lesions n (%)	Malignant n (%)	Nonmalignant n (%)	Cancer yield* (%)
Core needle biopsy	241 (57.5)	117 (69.9)	124 (49.4)	48.5
Ultrasound-guided ROLL excision	76 (18.1)	18 (10.7)	58 (23.1)	23.7
Mammography-guided vacuum-assisted biopsy	52 (12.4)	30 (17.9)	22 (8.8)	57. 7
Ultrasound-guided fine-needle aspiration and follow-up	36 (8.6)	1 (0.6)	35 (13.9)	2.8
MRI-guided radioguided occult lesion localization excision	7 (1.7)	0 (0.0)	7 (2.8)	0.0
MRI-guided vacuum-assisted biopsy	6 (1.4)	1 (0.6)	5 (2.0)	16.7
Surgical excision†	1 (0.2)	1 (0.6)	0 (0.0)	100.0
Total	419	168	251	40.1

* Cancer yield = number of malignancies per method.

† No previous localization method or biopsy mentioned in the electronic report.

ROLL, radioguided occult lesion localization.

Two hundred and thirty-four findings (55.8%) corresponded to masses, followed by 169 (40.3%) that were non-mass enhancements, eight (1.9%) that were suspicious lymph nodes, four (1.0%) that were foci, two (0.5%) that were fluid collections or abscesses, one (0.2%) that was a cystic lesion, and one (0.2%) that was described as a peri-implant fluid collection with peripheral enhancement. Out of the 168 malignancies, 99 (58.9%) appeared as masses, 68 (40.5%) as non-mass enhancements, and one (0.6%) as a suspicious lymph node. There was no relevant difference between masses and non-masses (excluding foci) regarding malignant outcomes (chi-square, 0.173; *p* = 0.683). The other descriptors were not linked to cancers in this study.

### BI-RADS 4 stratification

Of the 419 BI-RADS 4 lesions evaluated, 169 (40.3%) were subcategorized as BI-RADS 4A (low suspicion for malignancy), 136 (32.5%) as BI-RADS 4B (moderate suspicion for malignancy) and 114 (27.2%) as BI-RADS 4C (high suspicion for malignancy). Of the 169 BI-RADS 4A lesions, 24 (14.2%) were confirmed malignancies, compared with 56 (41.2%) of the 136 BI-RADS 4B lesions and 88 (77.2%) of the 114 BI-RADS 4C lesions. The malignancy probability was significantly different among the BI-RADS 4 subcategories (chi-square, 112,563; *p* < 0.001). Multiple comparisons between subcategories (4A vs. 4B; 4B vs. 4C; and 4A vs. 4C) also showed significant differences, even after Bonferroni correction (*p* < 0.001 for all).

**Figure 6:**
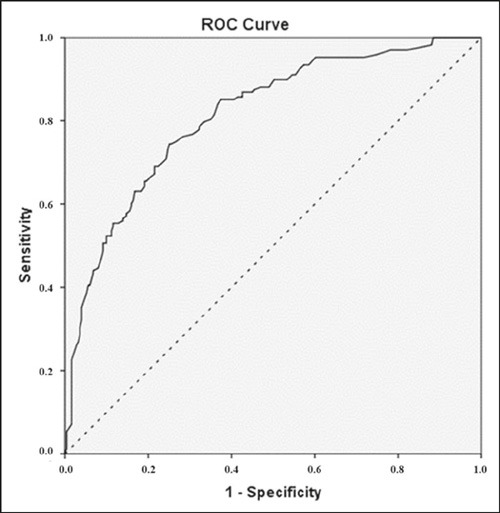
Equivalence/noninferiority graph showing the PPV2s and 95% CIs for BI-RADS 4 MRI subcategories (size of squares are proportional to the outcome number in each subcategory). For subcategories 4B and 4C, the PPV2s and respective confidence bounds are within the limits established for mammography and ultrasound, and are deemed equivalent (as highlighted in gray and darker gray, respectively). The PPV2 for subcategory 4A is above the 10% limit, although its lower confidence bound crosses this benchmark (as shown in lighter gray).

The PPV2 and 95% CIs for subcategories 4B and 4C were within the equivalence/noninferiority margins considered (Table 3). However, subcategory 4A had a PPV2 outside of the benchmarks established (PPV2, 14.2%; 95% CI: 9.3–20.4%), as can be seen in Figure 6. Although the lower 95% CI bound extends below the 10% limit, BI-RADS 4A should be considered nonequivalent.

**Table 3:** Probability of malignancy for stratified suspicious lesions (BI-RADS 4 subcategories).

MRI criteria	Examinations (n)	Malignancies (n)	PPV2*	95% CI†	Established benchmarks‡
BI-RADS 4 subcategory					
4A	169	24	14.2	9.3–20.4	> 2 to ≤ 10
4B	136	56	41. 2	32.8–49.9	> 10 to ≤ 50
4C	114	88	77. 2	68.4–84.5	> 50 to < 95
Total	419	168	40.1	35.4–45.0	—

* Based on the recommendation for tissue diagnosis, according to the ACR BI-RADS.

† Calculated by the Clopper-Pearson method.

‡ Probability range for malignancy recognized for mammography and ultrasound in the ACR BI-RADS.

### Predictive model

Univariate analysis of each of the studied variables showed significant results, allowing their inclusion in the multivariate model. All of them had an *omnibus p* < 0.001. Equivalent *p*-values were also found for the individual strata of the categorical variables, as shown in Table 4.

**Table 4:** Univariate and multivariate logistic regression analyses for risk stratification of suspicious (BI-RADS 4) lesions by MRI criteria.

Predictive variables	β coefficient	OR	95% CI	P
Univariate models				
Age	0.034	1.035	1.018–1.052	< 0.001
BI-RADS 4 subcategory				< 0.001
4A	0.000	1.000*		
4B	1.442	4.229	2.439–7.335	
4C	3.018	20.449	11.058–37.815	
Multivariate model				< 0.001
Age	0.033	1.033	1.014–1.053	0.001
BI-RADS 4 subcategory				< 0.001
4A	0.000	1.000*		
4B	1.364	3.913	2.236–6.846	
4C	2.997	20.021	10.738–37.329	

* Reference value.

**Figure 7:**
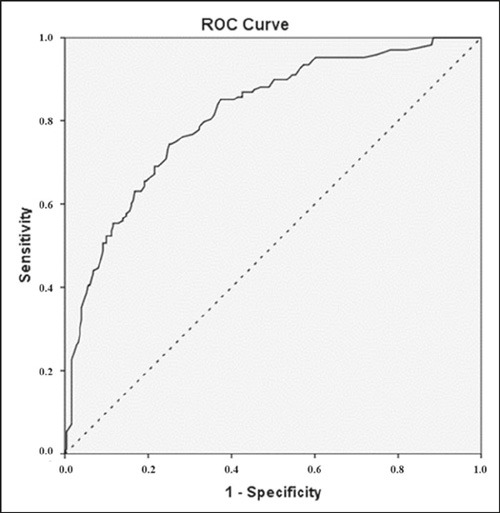
ROC curve from the full model including BI-RADS 4 subcategories and patient ages (continuous line) has an AUC of 0.813 (95% CI: 0.772–0.855). The dashed line represents the reference.

The predictors were included in a backward stepwise approach in two steps, each attaining statistical significance by the chi-square test (*p* < 0.001). The full model contained age and the BI-RADS 4 subcategories (*p* = 0.001 and *p* < 0.001, respectively), with the odds of cancer being highest for subcategory 4C (OR, 20.021; 95% CI: 10.738–37.329), as demonstrated in Table 4. The goodness-of-fit of the model was considered acceptable, as evidenced by the Nagelkerke’s R^2^ (0.364) and the nonsignificant Hosmer-Lemeshow *p* value (*p* = 0.814), with an overall predictive performance of 74.5% (nonmalignant, 83.7%; malignant, 60.7%). Finally, the ROC curve generated from this model produced an AUC of 0.813 (95% CI: 0.772–0.855; *p* < 0.01) indicating good diagnostic discrimination (Figure 7).

## DISCUSSION

The main objective of this study was to prove that the malignancy probability observed after stratification of category 4 findings detected on MRI would be equivalent to that established for mammography and ultrasound in the ACR BI-RADS. We employed an equivalence/nonin-feriority statistical approach and demonstrated that the subcategorization might be achieved in real-world clinical practice. The PPV2 calculated for subcategories 4B and 4C determined by MRI criteria were within the established margins indicating equivalence. However, the PPV2 for subcategory 4A was slightly above the upper margin of equivalence.

The malignancy probability range accepted by the ACR for BI-RADS category 4 lesions observed on mammography and ultrasound is quite large (> 2% and ≤ 95%). Because the recommendation for suspicious findings is tissue sampling, a relatively high number of negative biopsies can be expected, which might pose a problem in further patient management and decrease the cost-effectiveness of screening with MRI. Therefore, the stratification of category 4 into more manageable subcategories not only plays an important role in auditing practices but also influences the decision-making process of attending physicians. Unfortunately, because of the paucity of published data, MRI stratification is not yet officially recommended^(27)^. In addition, a higher baseline risk is anticipated for most patients undergoing breast MRI, regardless of the screening or diagnostic context^(20,21,22)^. Therefore, it would not come as a surprise if the cancer yield associated with BI-RADS 4 MRI findings was higher than the probability ranges already stipulated for the other imaging methods. To our knowledge, this is the first study to apply equivalence/non-inferiority statistical standards to the stratification of suspicious findings by MRI criteria.

In another retrospective study, Strigel et al.^(28)^ concluded that the stratification of category 4 lesions on MRI was feasible and met the probability ranges specified for mammography and ultrasound. However, in that study, subcategories 4A and 4C, despite presenting PPV2 results well within the stipulated ranges for each stratum, showed 95% CIs that crossed the accepted margins. That would lead to the conclusion that there was non-similarity by equivalence/noninferiority statistical norms. Maltez de Almeida et al.^(23)^ also reported large 95% CIs and a higher PPV2 for subcategory 4A, in accordance with the findings of the present study. In contrast, Honda et al.^(29)^ reported a below-threshold PPV of 1.8% for low-suspicion lesions (subcategory 4A), with wide-ranging 95% CIs. In a meta-analysis, Li et al.^(30)^ not only showed high heterogeneity across the selected studies but also corroborated our finding that the malignancy ranges for the MRI subcategories are larger than the those recommended in the ACR BI-RADS, which are as follows: 4A, low suspicion (> 2% but ≤ 10%); 4B, moderate suspicion (> 10% but ≤ 50%) and 4C, high suspicion (> 50% but < 95%). In accordance with an expected higher pre-test probability of malignancy in patients submitted to breast MRI, the authors of that meta-analysis reported that the upper range reached 18.3% for subcategory 4A, 57.5% for subcategory 4B, and 95.2% for subcategory 4C. The data indicate that it is indeed feasible to stratify category 4 MRI findings, although the malignancy probability range might be wider than what is accepted for the other imaging methods^(23,28,29,30)^.

Univariate and multivariate analyses further supported the relevance of dynamic contrast-enhanced MRI criteria for malignancy risk stratification in suspicious lesions. The ROC curve generated from the probabilities derived from the full model showed good accuracy, albeit lower than that reported previously^(23)^. That could be explained, at least in part, by the retrospective nature of our study, which accounted for a real-world clinical practice scenario in which variability of image interpretation and technical issues might be a considerable source of bias.

Our study has some other limitations. It was conducted at a single center and employed retrospective analysis of data from an electronic database, which could have introduced an unintended patient selection bias. Our center is a private facility, and, although all of the interpreters were experienced radiologists specializing in breast imaging, the results obtained might be less optimal than those obtained in studies conducted at large academic centers with state-of-the-art equipment. We purposefully did not factor the indication for MRI into our analyses. As a result, diagnostic studies were mixed with those designated as screening studies, which would be expected to increase the malignancy ratio in our sample. Despite being considered a limitation, this approach was intended to better represent daily practice at many private centers worldwide, in which a considerable number of indications are either unclear or not in accordance with consensus recommendations.

Another relevant aspect is the impact of additional imaging methods on the PPV of MRI findings^(31,32)^. It has been shown that lesion detection, particularly by second-look ultrasound, is directly related to the type of enhancement (mass or non-mass) and varies widely^(31)^. That issue was not directly addressed here, because it was outside the scope of this study. Nevertheless, we recognize the importance of the subject and hope that further studies will provide greater insights into the topic.

The lack of ACR BI-RADS guidelines for the stratification of category 4 lesions leads to subjectivity in their subcategorization. Few of the studies on the topic have provided a clear list of parameters employed for stratification. Therefore, “personal experience” must be considered along with more objective criteria. We tried to account for subjective judgment and the broader clinical context, considering some overlap in the number of descriptors permitted for subcategories 4A and 4B. However, we understand that our solution might not accommodate all of the particularities observed in daily practice.

## CONCLUSION

Stratification of BI-RADS assessment category 4 by MRI criteria is feasible in real-world clinical practice. Nevertheless, malignancy probability ratios higher than those observed for mammography and ultrasound might be encountered. Larger studies are needed in order to evaluate the malignancy probabilities related to individual imaging characteristics and MRI descriptors, indicating which are better fits for each BI-RADS 4 subcategory.
